# Supplementary material for: Cross-Linking of Doped Organic Semiconductor Interlayers for Organic Solar Cells: Potential and Challenges
Source: ACS Appl Energy Mater. 2021 Dec 10;4(12):14458–66. doi: 10.1021/acsaem.1c03127 (PMC8715538; doi:10.1021/acsaem.1c03127)
Supplement: Supplementary file 1 — ae1c03127_si_001.pdf [file ae1c03127_si_001.pdf]

Supporting Information: Cross-Linking of Doped Organic Semiconductor Interlayers for Organic Solar Cells: Potential and Challenges

Staffan Dahlström,*,† Sebastian Wilken,† Yadong Zhang,‡,¶ Christian Ahläng,† Stephen Barlow,‡,¶ Mathias Nyman,† Seth R. Marder,‡,¶,§,‖ and Ronald Österbacka†

†*Physics, Faculty of Science and Engineering, Åbo Akademi University, Henriksgatan 2, 20500 Turku, Finland*

‡*School of Chemistry & Biochemistry, Georgia Institute of Technology, Atlanta GA 30332, United States*

¶*Renewable and Sustainable Energy Institute, University of Colorado Boulder, Boulder CO 80303, United States*

§*Department of Chemical and Biological Engineering, University of Colorado Boulder, Boulder CO 80303, United States*

‖*Department of Chemistry, University of Colorado Boulder, Boulder CO 80303, United States*

* E-mail: stdahlst@abo.fi

Synthesis of the Cross-Linker

The crosslinker has previously been obtained from the reaction of 4-azido-2,3,5,6-tetrafluoro-benzoyl chloride and benzene-1,3,5-triol.[S1] Here we used a different route, as shown Figure S1, the intermediate benzene-1,3,5-triyl tris(2,3,4,5,6-pentafluorobenzoate) being synthesized in a similar way to a previous report.[S2]

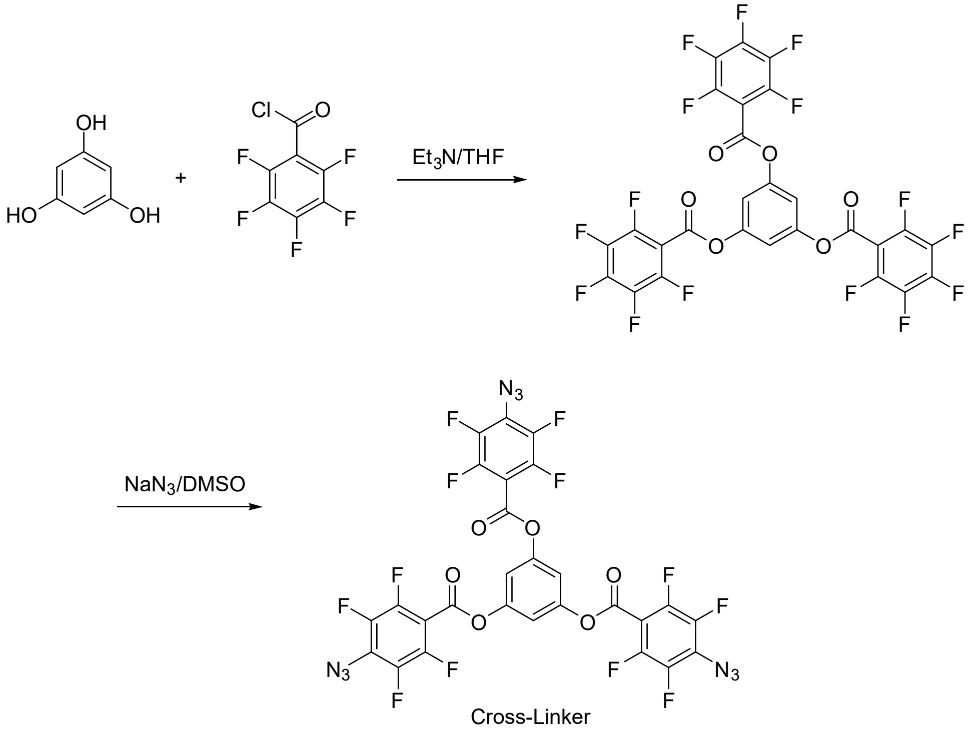

Figure S1: Synthesis of the cross-linker used in this work.

Benzene-1,3,5-triyl tris(2,3,4,5,6-pentafluorobenzoate). A solution of benzene-1,3,5-triol (1.8 g, 14.3 mmol) in THF (25 mL) and Et_3N (4 mL) was added over 30 min to a solution of 2,3,4,5,6-pentafluorobenzoyl chloride (10.0 g, 43.4 mmol) in THF (25 mL) at room temperature under nitrogen. The reaction mixture was stirred at room temperature for 5 h. THF was removed under reduced pressure and water (100 mL) was added, resulting in the appearance of a pink solid, which was collected by filtration, and purified through column chromatography on silica gel using dichloromethane/hexane (1:1) as the eluent. After removal of solvents

and drying under vacuum, the pure product was obtained as white solid (5.2 g, 52%). ^{1}H NMR (400 MHz, $CDCl_3$) δ: 7.29 (s, 3H) ppm. ^{19}F NMR (376.5 MHz, $CDCl_3$) δ: -136.37 (m, 2F), -145.67 (m, 1F), -159.33 (m, 2F) ppm.

Benzene-1,3,5-triyl tris(4-azido-2,3,5,6-tetrafluorobenzoate) (Cross-Linker). A solution of NaN_3 in a mixture of DMSO and H_2O (5:1 v/v, 6.0 mL) was added over 6 h to a solution of benzene-1,3,5-triyl tris(2,3,4,5,6-pentafluorobenzoate) (0.50 g, 0.71 mmol) in a mixture of DMSO and THF (1:1 v/v, 10 mL) at room temperature. The reaction mixture was stirred at room temperature for 22 h; water (100 mL) was then added; the resulting pink solid was collected by filtration and washed with methanol. The product was purified through column chromatography on silica gel using dichloromethane/hexane (3:2) as the eluent. After removal of solvents under reduced pressure and drying under vacuum, the pure product was obtained as a white solid (0.40 g, 73%). ^{1}H NMR (400 MHz, $CDCl_3$) δ: 7.26 (s, 3H) ppm. ^{19}F NMR (376.5 MHz, $CDCl_3$) δ: -137.00 (m, 2F), -150.23 (m, 2F) ppm.

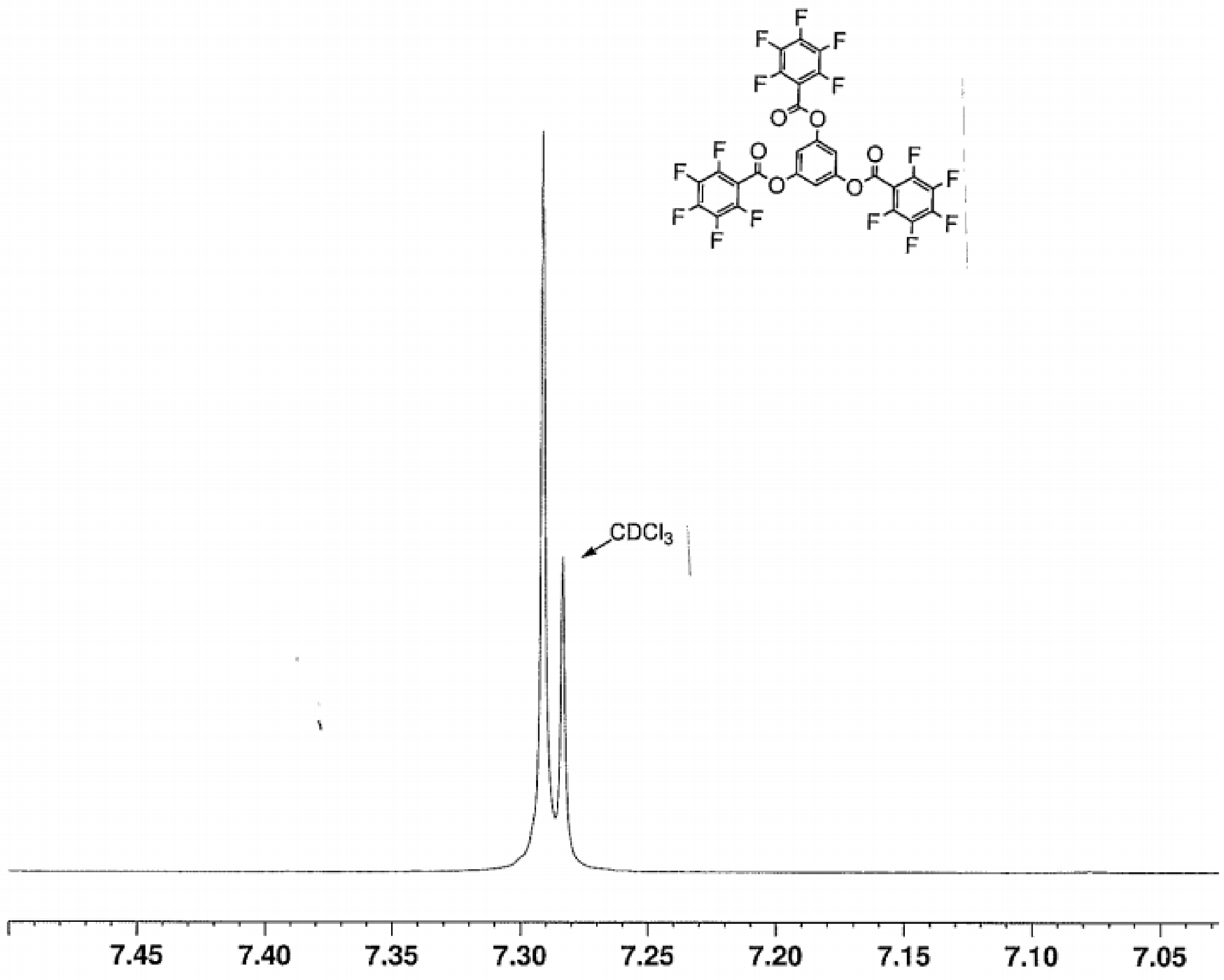

Figure S2: ^{1}H spectrum for benzene-1,3,5-triyl tris(2,3,4,5,6-pentafluorobenzoate).

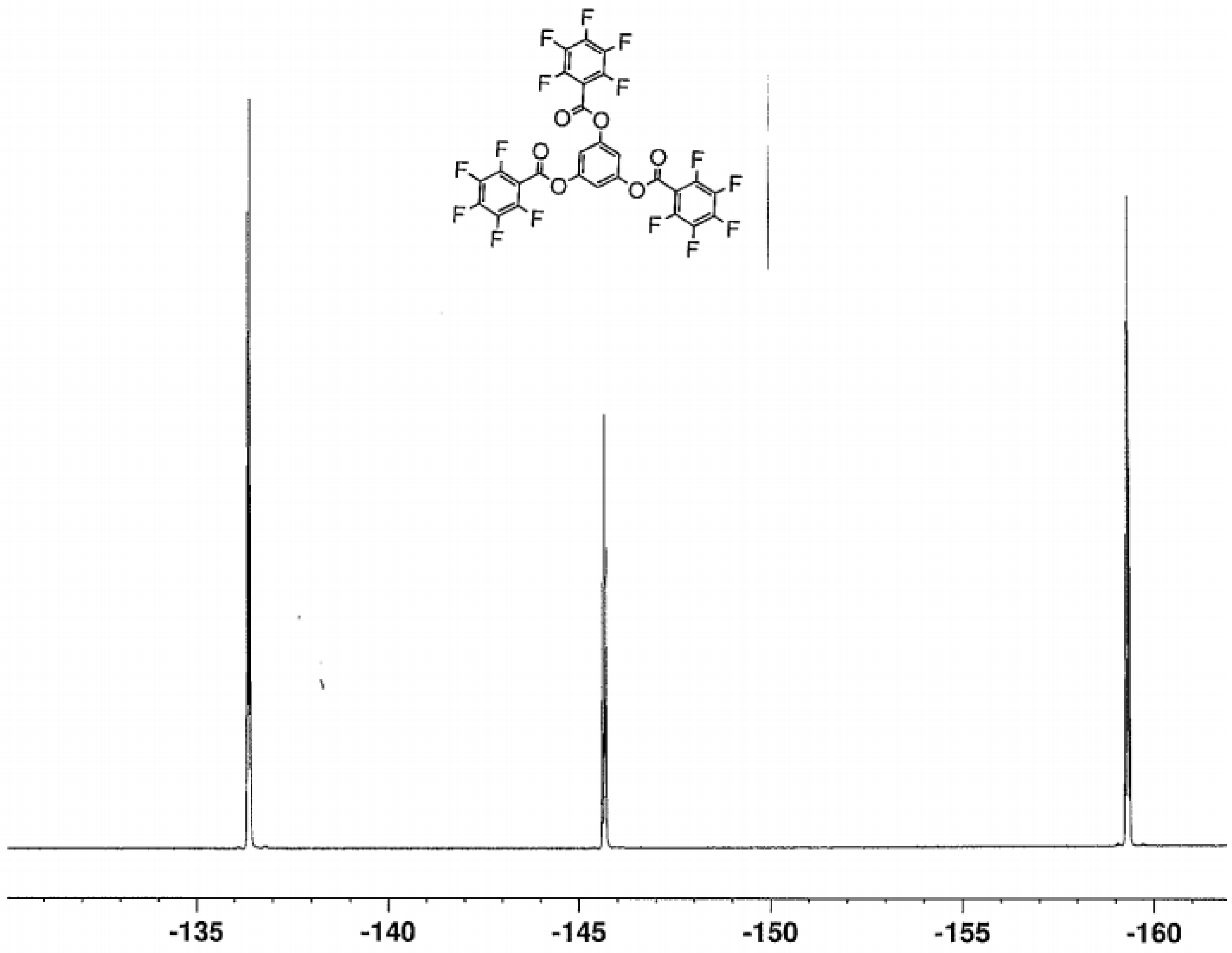

Figure S3: ^{19}F spectrum for benzene-1,3,5-triyl tris(2,3,4,5,6-pentafluorobenzoate).

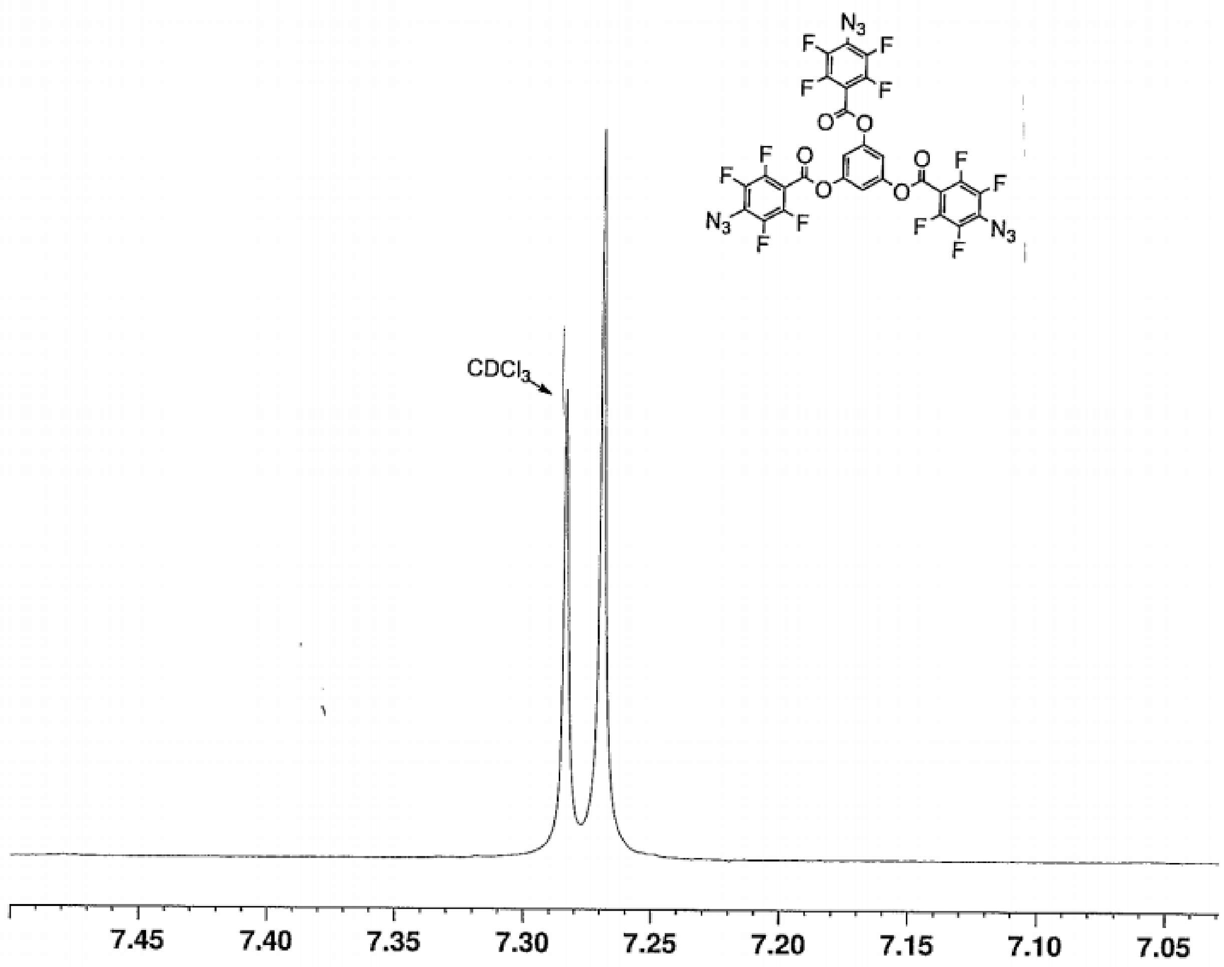

Figure S4: ^{1}H spectrum for benzene-1,3,5-triyl tris(4-azido-2,3,5,6-tetrafluorobenzoate).

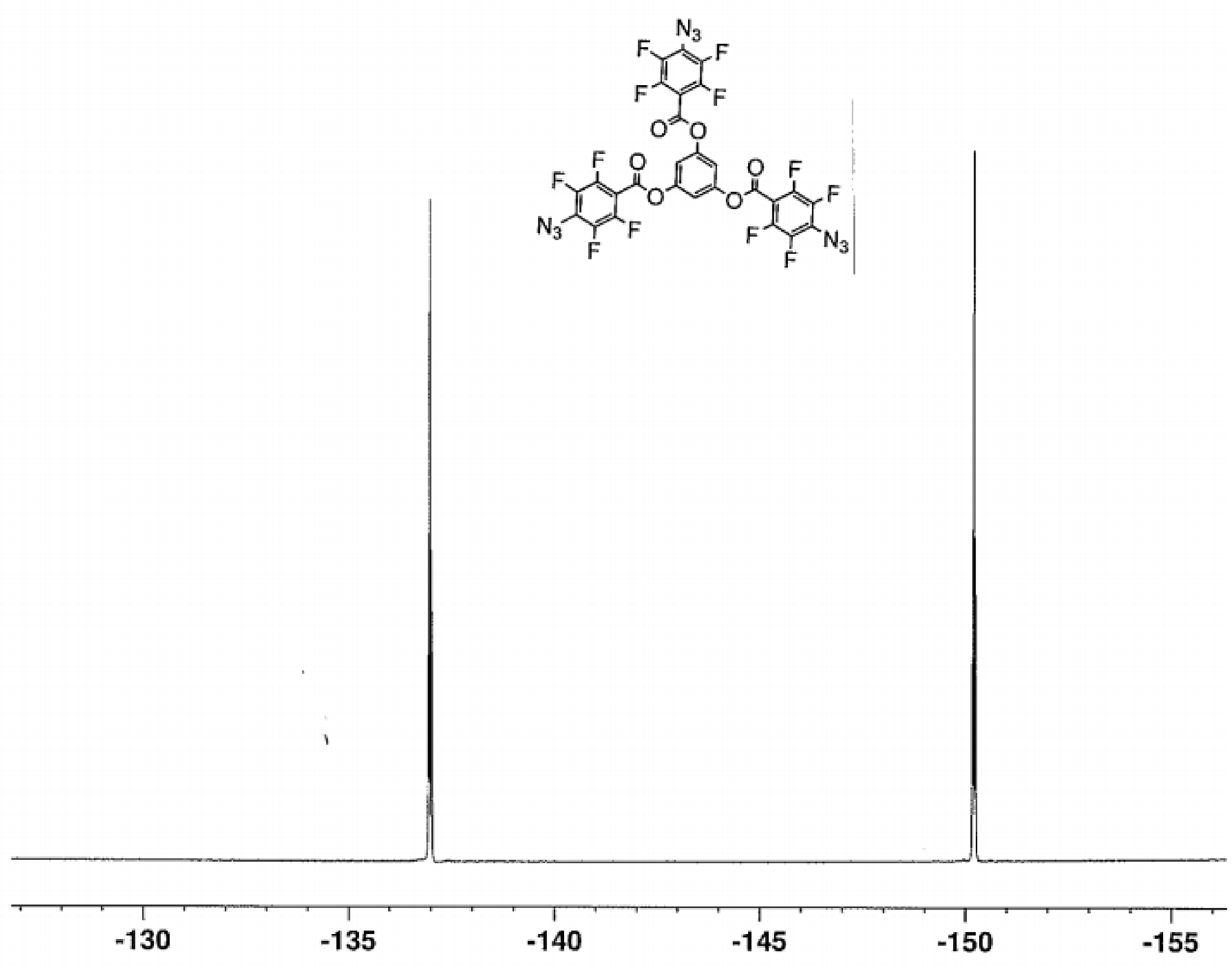

Figure S5: ^{19}F spectrum for benzene-1,3,5-triyl tris(4-azido-2,3,5,6-tetrafluorobenzoate).

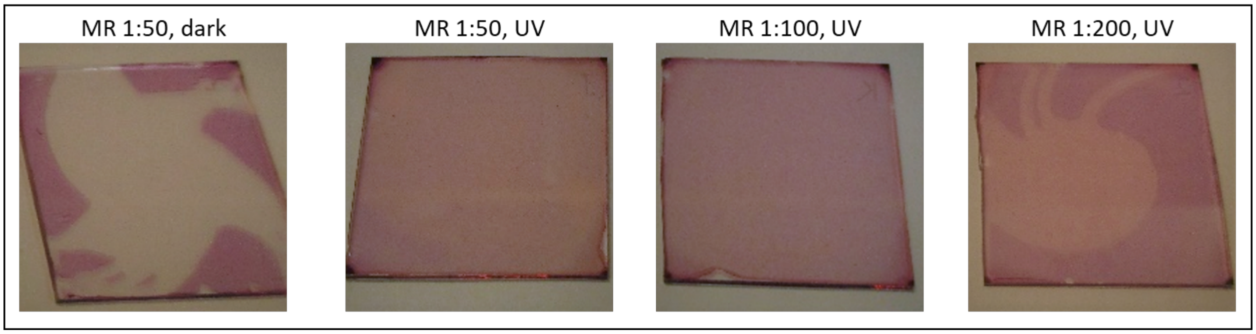

Figure S6: Photographs of thin films of P3HT with the cross-linker added in different molar ratio (MR) after rinsing with 1,2-dichlorobenzene. The left photo shows a film kept in the dark, while the films in the other three photos were treated with UV light to initialize the cross-linking mechanism.

Transfer-Matrix Model

To calculate the spatial generation rate profile $G(x)$ in the active layer, optical transfer-matrix simulations were performed using a MATLAB code.[S3] The optical constants of all involved material layers were taken from spectroscopic ellipsometry measurements published in previous reports.[S4–S6] Here, we made the assumption that the introduction of the dopant and the cross-linker has only negligible effect on the optical properties of the P3HT used as HTL, which is reasonable given the low weight percentage of the impurities. Figure S7 shows the generation rate profile for a device with a HTL of P3HT and PEDOT:PSS, respectively. While the type of HTL has virtually no effect on the shape of $G(x)$, its magnitude is significantly higher for the PEDOT:PSS device. The reason is that PEDOT:PSS absorbs less light in the visible spectrum, i.e., shows a higher transparency. The generation profiles were used to calculate the generation current J_G, that is, the maximum achievable photocurrent if there were no conversion and collection losses, via

$$J_G = q \int_0^L G(x)\,\mathrm{d}x, \tag{1}$$

where q is the elementary charge and L is the active-layer thickness.

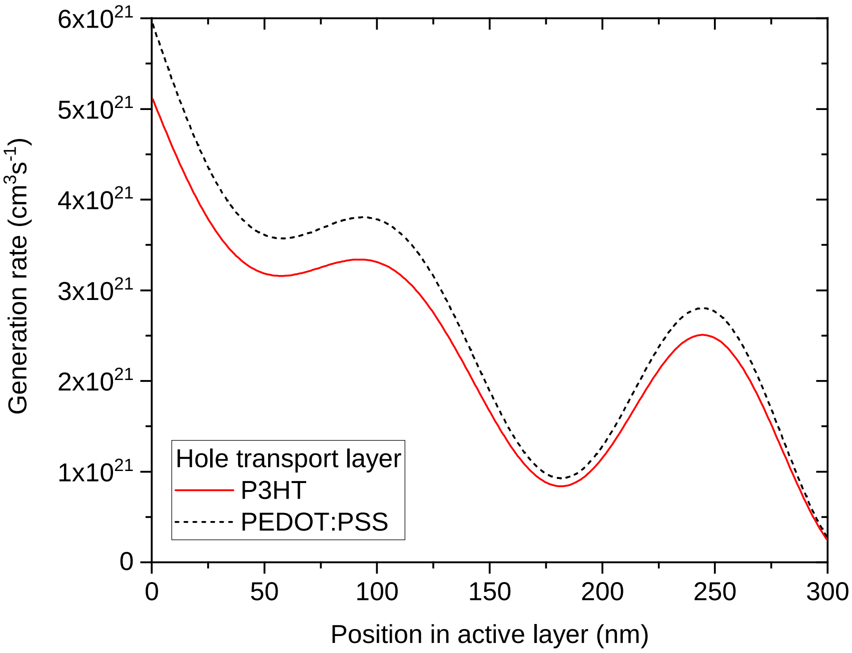

Figure S7: Spatial generation profile $G(x)$ for a P3HT:PCBM solar cell with a hole transport layer of doped P3HT and PEDOT:PSS, respectively.

Photovoltaic Performance

A summary of the photovoltaic parameters open-circuit voltage (V_{OC}), short-circuit current (J_{SC}), fill factor (FF), and power conversion efficiency (PCE) for the reference device with a PEDOT:PSS interlayer and the devices with a cross-linked P3HT layer at various doping concentrations is presented in Table S1.

Table S1: Performance of P3HT:PCBM solar cells with different interlayers.

Interlayer	V_{OC} (V)	J_{SC} (mA cm^{-2})	FF (%)	PCE (%)
PEDOT:PSS	0.61	7.6	64	3.0
P3HT, 0 wt%	0.50	1.2	27	0.2
P3HT, 2 wt%	0.55	6.9	32	1.2
P3HT, 4 wt%	0.58	6.9	43	1.7
P3HT, 6 wt%	0.56	6.4	43	1.5
P3HT, 10 wt%	0.55	4.3	47	1.1

CELIV Measurements

Exemplary CELIV transients for solar cells with PEDOT:PSS or cross-linked P3HT at various dopant concentrations as HTL are shown in Figure S8. All measurements were performed at an voltage pulse amplitude of 2 V, an offset voltage of $V_{\text{off}} = 0\,\text{V}$ and a pulse length of 500 μs. The measured current is normalized to j_0 and plotted versus time t. For the samples with PEDOT:PSS and undoped P3HT, the transient current $j(t)$ equals the current j_0 given by the geometric capacitance of the device. In other words, both samples are effectively undoped. For the samples with a doped P3HT interlayer, there is an additional time dependent extraction current $\Delta j(t)$ caused by the extraction of doping-induced charge carriers from the active layer. For the doping-CELIV measurements the pulse length was varied to ensure that the measurements are performed in the doping-induced capacitive regime. This regime is reached at long enough pulse lengths where the transient is determined by the width of the space charge region caused by doping, $j(t) = \varepsilon\varepsilon_0 A w_{\text{SCR}}^{-1}$, and transients in this regime therefore overlap, see Figure S9.

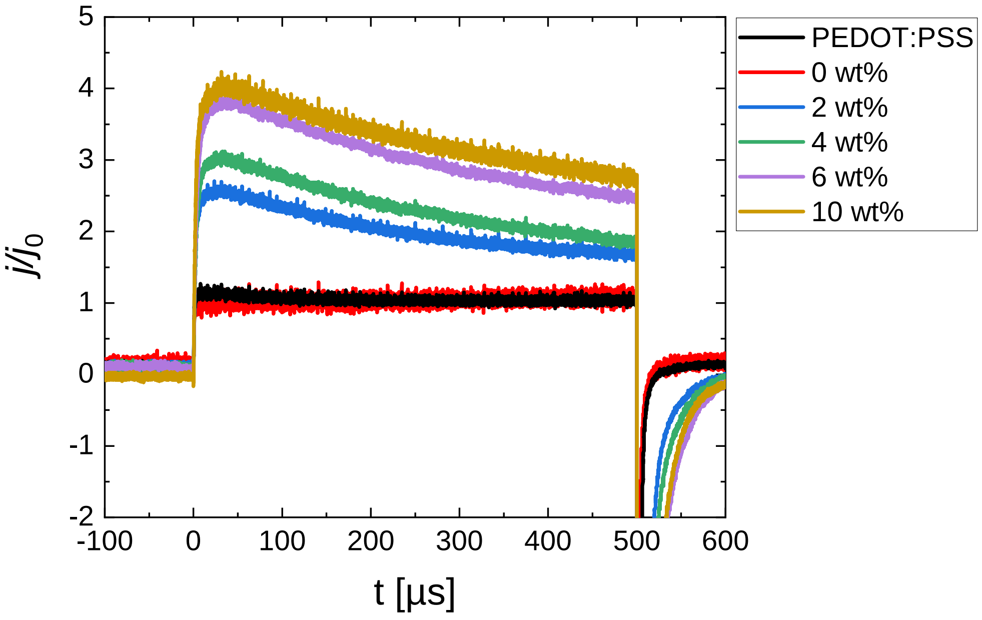

Figure S8: CELIV transients for P3HT:PCBM solar cells with different HTLs.

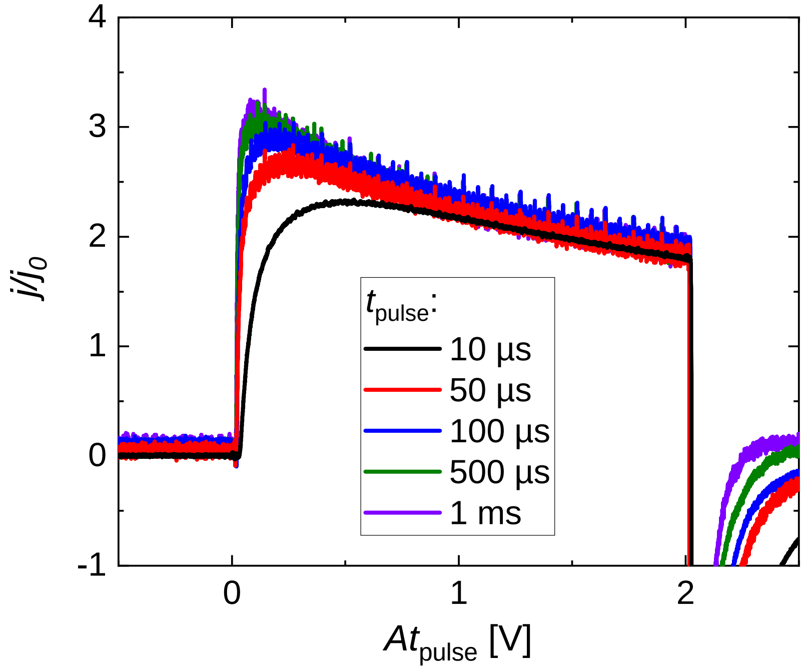

Figure S9: CELIV transients at various pulse lengths, for a P3HT:PCBM solar cell with an interlayer doped at 4 wt%, demonstrating the doping induced capacitive regime reached at longer pulse lengths where the transients overlap. The amplitude of the applied voltage pulse is 2 V and the applied offset voltage is 0 V.

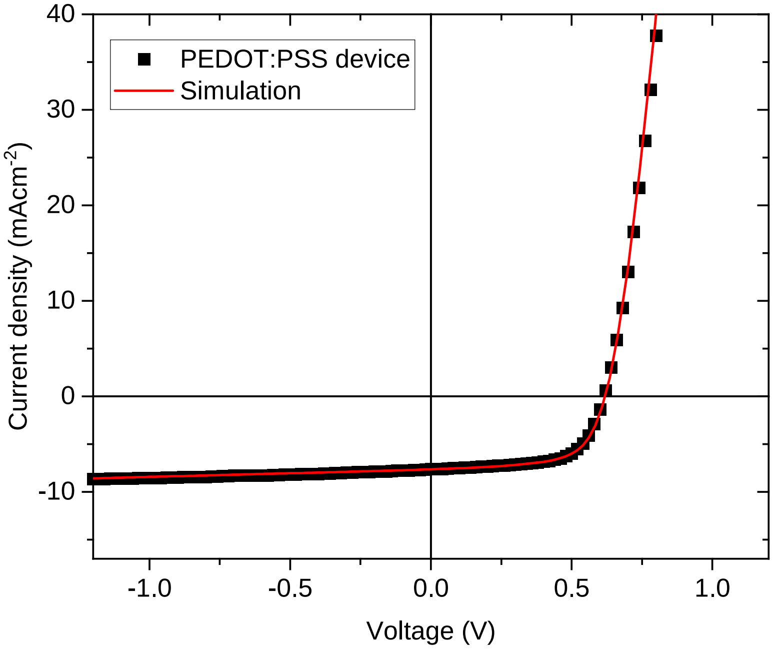

Figure S10: Measured and modeled current–voltage curves for a P3HT:PCBM solar cell with a HTL of PEDOT:PSS. The parameters used in the numerical drift–diffusion model are given in Table 2 in the main text. The generation rate was taken from the transfer-matrix model, see Figure S7, and its spatial dependence was fully accounted for.

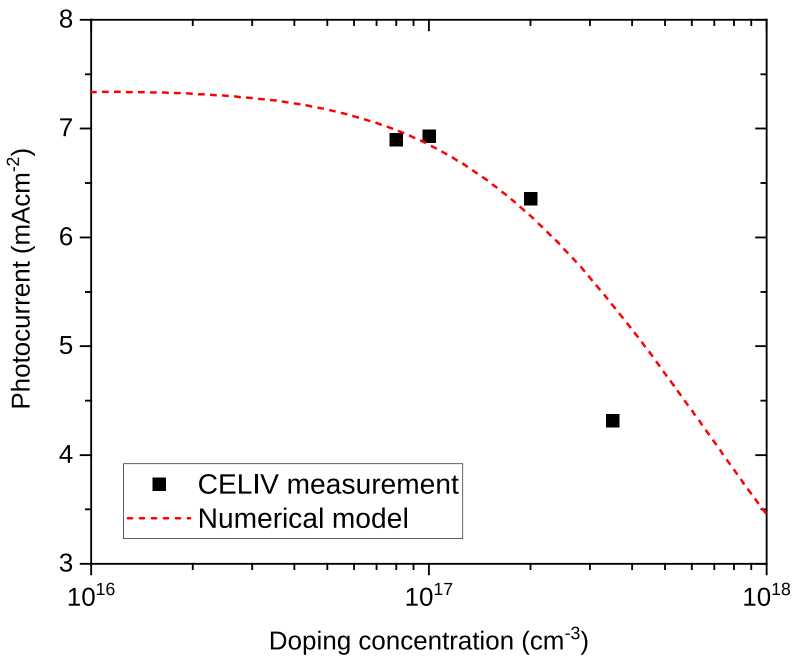

Figure S11: Measured and modeled dependence of the photocurrent (shown is the short-circuit current J_{SC}) on the doping concentration in the active layer. Experimental values were taken from the doping-CELIV measurements shown in the main text. The discrepancy between experiment and model at high doping concentrations suggest that the real doping profiles are not constant as assumed in the model, but show a more complex spatial dependence with an increased concentration towards the anode.

References

(S1) Tang, B.; Wu, Y.; Tai, A.; Hu, R.; Zhao, Z.; Shenqing, F. Z. Patent CN 105906807 A. 2016.

(S2) Demyanov, P. I.; Jimenez, M. P.; Bogdashkina, V. I.; Petrosyan, V. S. Synthesis, stability and chromatographic properties of pentafluorobenzoates of phenols. *Vestnik Moskovskogo Universiteta Seriya 2: Khimiya* **1991**, *32*, 180.

(S3) Burkhard, G. F.; Hoke, E. T.; McGehee, M. D. Accounting for Interference, Scattering, and Electrode Absorption to Make Accurate Internal Quantum Efficiency Measurements in Organic and Other Thin Solar Cells. *Adv. Mater.* **2010**, *22*, 3293–3297.

(S4) Wilken, S.; Wilkens, V.; Scheunemann, D.; Nowak, R.-E.; von Maydell, K.; Parisi, J.; Borchert, H. Semitransparent Polymer-Based Solar Cells with Aluminum-Doped Zinc Oxide Electrodes. *ACS Appl. Mater. Interfaces* **2015**, *7*, 287–300.

(S5) Scheunemann, D.; Wilken, S.; Parisi, J.; Borchert, H. Investigation of the Spatially Dependent Charge Collection Probability in $CuInS_2$/ZnO Colloidal Nanocrystal Solar Cells. *ACS Photonics* **2015**, *2*, 864–875.

(S6) Wilken, S.; Sandberg, O. J.; Scheunemann, D.; Österbacka, R. Watching Space Charge Build Up in an Organic Solar Cell. *Sol. RRL* **2020**, *4*, 1900505.
